# Post-mastectomy radiotherapy benefits subgroups of breast cancer patients with T1–2 tumor and 1–3 axillary lymph node(s) metastasis

**DOI:** 10.2478/raon-2013-0085

**Published:** 2014-07-10

**Authors:** Yu-Li Su, Shan-Hsuan Li, Yen-Yang Chen, Hui-Chun Chen, Yen Tang, Cheng-Hua Huang, Fong-Fu Chou, Shih-Chung Wu, Kun-Ming Rau

**Affiliations:** 1 Division of Hematology-Oncology, Department of Internal Medicine, Kaohsiung Chang Gung Memorial Hospital, Kaohsiung, Taiwan; 2 Chang Gung University, College of Medicine, Taoyuan, Taiwan; 3 Department of Radiation Oncology, Kaohsiung Chang Gung Memorial Hospital, Kaohsiung, Taiwan; 4 Division of Hematology-Oncology, Department of Internal Medicine, E-Da Hospital, Kaohsiung, Taiwan; 5 Department of General Surgery, Kaohsiung Chang Gung Memorial Hospital, Kaohsiung, Taiwan; 6 Cancer Center, Kaohsiung Chang Gung Memorial Hospital, Kaohsiung, Taiwan

**Keywords:** breast cancer, postmastectomy radiotherapy, overall survival, locoregional recurrence, lymphovascular invasion

## Abstract

**Background:**

To determine the role of postmastectomy radiotherapy (PMRT) in breast cancer patients with T1–2 and N1 disease.

**Patients and methods.:**

A total of 207 postmastectomy women were enrolled. The 5-year Kaplan-Meier estimates of locoregional recurrence rate (LRR), distant recurrence rate (DRR) and overall survival (OS) were analyzed by different tumor characteristics. Multivariate analyses were performed using Cox proportional hazards modeling.

**Results:**

With median follow-up 59.5 months, the 5-year LRR, DRR and OS were 9.1%, 20.3% and 84.4%, respectively. On univariate analysis, age < 40 years old (p = 0.003) and Her-2/neu over-expression (p = 0.016) were associated with higher LRR, whereas presence of LVI significantly predicted higher DRR (p = 0.026). Negative estrogen status (p = 0.033), Her-2/neu overexpression (p = 0.001) and LVI (p = 0.01) were significantly correlated with worse OS. PMRT didn’t prove to reduce 5-year LRR (p = 0.107), as well as 5-year OS (p = 0.918). In subgroup analysis, PMRT showed significant benefits of improvement LRR and OS in patients with positive LVI.

**Conclusions:**

For patients with T1–2 and N1 stage breast cancer, PMRT can decrease locoregional recurrence and increase overall survival only in patients with lymphovascular invasion.

## Introduction

Treating breast cancer patients often requires a multidisciplinary approach. The standard treatment is resection of primary breast tumor with axillary lymph nodes dissection, and adjuvant therapies such as chemotherapy, hormonal therapy or post-mastectomy radiotherapy (PMRT) should be done guided by clinicopathologic factors. Adjuvant radiotherapy is indicated for patients who undergo breast-conserving surgery (BCS). For patients who received total mastectomy, there are also many studies demonstrated that PMRT reduced locoregional recurrence (LR) as well as improved disease free survival (DFS) and overall survival (OS).^1^–^7^ Although results from the Early Breast Cancer Trialists’ Cooperative Group (EBCTCG) showed that benefits of PMRT were emerged in all patients with positive lymph nodes (LN) 8, the guideline of American Society of Clinical Oncology recommends adjuvant radiotherapy is only suggested for patients who received BCS or total mastectomy with T3 or more than three (N2) positive axillary LN.^9^ In St. Gallen Consensus Conference 2011, routine PMRT was clearly endorsed for patients with more than 3 involved nodes (88% yes, 5% no), but was reduced for patients with 1–3 affected nodes (18% yes, 71% no), unless if young patients (< 45 years of age; 51% yes, 42% no) or presented with extensive vascular invasion (57% yes, 26% no).^10^ Even in the guideline of National Comprehensive Cancer Network (NCCN), PMRT is still not routinely suggested for 1–3 positive LN patients. Therefore, for patients with T1–2 tumors and 1–3 positive LN, there is much of controversy whether PMRT has significant survival benefit, especially the side effects from radiation always happen during or after the course of radiotherapy.

There are existing evidences proved that PMRT and adjuvant chemotherapy significantly improve locoregional DFS in N1 breast cancer patients.^1^–^4^,^11^,^12^ The Danish 82b & 82c and British Columbia trials showed survival benefits from PMRT in both patients with 1–3 versus 4 or more positive LN.^1^–^4^ On the contrast, McArdle *et al*. presented that a significant advantage in cancer specific survival afforded by PMRT was seen only in patients with ≥ 4 positive nodes.^12^ The discrepancy may be partly because suboptimal dissection of axillary LN in the Danish Trials (median, 7 nodes), compared with other similar series (median, 15 nodes) 13–16, and it resulted in higher locoregional failure rate in the subgroup (1–3 LN without PMRT) of the Danish Trials (30%) compared with others (15%).^13^–^16^ The recent analysis, selecting patients from the Danish study with 8 or more nodes removed concluded that the 15-year absolute magnitude of survival benefit was 9% in patients with either 1–3 or 4 or more positive LN.^17^

Several retrospective series tried to determine predictive and risk factors of recurrence among this subgroup. Age < 45 years old, more than 25% positive node ratio, medial tumor location, estrogen receptor (ER) negative status and lymphovascular invasion (LVI) all are independently significant factors of LR.^18^–^20^ Multidisciplinary therapy, including PMRT, should be considered to apply in this subgroup for optimal local control and possible survival benefit. Subsequent studies need to identify the risk factors of LR, in order to clarify actual benefits from PMRT in different subgroups.

The aim of our study is to find the predictive markers of the indication of PMRT in patients with T1–2 and 1–3 positive LN. In addition, for patients with known risk factors, we also examined the differences of LRR and OS whether PMRT was performed or not.

## Patients and methods

### Patients

With the permission from institutional review board, we retrospectively reviewed medical records of patients who were pathologically diagnosed with T1–T2 and N1 staged invasive breast cancer at the Kaohsiung Chang Gung Memorial Hospital between Jan. 2000 and Dec. 2006. A total of 207 patients received modified radical mastectomy (MRM) or simple mastectomy, with or without PMRT were included consecutively.

The basic characteristics of patients included age, histopathology, size of primary tumor (T1 or T2), numbers of removed and involved LN, LVI, ER, Her-2/neu status and types of systemic therapy. The location and timing of recurrence, together with date of death, were recorded to define locoregional recurrence (LR), distant recurrence (DR) and OS. LR was defined as recurrent tumors at residual breast, previous operation area, ipsilateral chest wall and clinical or radiographic proved lymphadenopathy over regional lymphatics (ipsilateral axillary, supraclavicular, internal mammary LN).

### Treatments

All patients underwent mastectomy with axillary LN dissection. External-beam irradiation was delivered with a total dose of 45–50.4 Gy in 25–28 fractions, and a subsequent 10–14 Gy boost to tumor bed if pathologically positive or close base margin (< 2mm). The fields of irradiation included tumor bed, chest wall, axillary and supraclavicular nodes. The internal mammary nodes were irradiated only if tumor located in medial side.

Adjuvant chemotherapy was chosen by clinicians in view of the characteristics of patients and tumors. The most commonly used regimens were anthracycline-based regimen and cyclophosphamide/methotrexate/5-fluorouracil (CMF). Patients with positive ER status would take at least 5-year of adjuvant endocrine therapy unless known contraindication or intolerance.

### Statistical analysis

To compare the clinicopathologic characteristics of tumors and patients between two study cohorts, we used Chi-square and Fisher’s exact test for categorical variables. The 5-year estimates of LRR, DRR and OS were computed by Kaplan-Meier methods and log-rank tests to determine statistic significance. Cox proportional hazard modeling was used for multivariate analysis of LRR, DRR and OS. Factors such as age (< 40 or ≥ 40), histopathologic types (invasive ductal carcinoma or others), primary tumor size, percentage of positive LN (< 25% or ≥ 25%), ER and Her-2/neu status and adjuvant chemotherapy or PMRT were all included as parameters. P value < 0.05 was considered as statistic significance, and all tests were two-tailed. All analyses were performed by PASW software version 18.1 (IBM & SPSS Inc., Somers, NY, USA).

## Results

### Patients and treatment characteristics

The clinicopathologic characteristics of study cohort were shown as Table 1. A total of 207 breast cancer patients who were pathologically diagnosed with T1–2 tumors and N1 status were enrolled. The median follow-up was 59.5 months, and mean age at diagnosis was 50.6 years. All patients received modified radical mastectomy or simple total mastectomy with axillary LN dissection. Of these patients, 35.7% (N = 74) and 64.3% (N = 133) patients were with T1 and T2 tumors, respectively. The percentage of ER positive and Her-2/neu overexpression were 66.2% and 20.5%. The mean number of removed LN was 15.4 (range, 2–38). Eighty-one patients (39%) received PMRT, which was decided by clinicians or combined conference. Adjuvant systemic therapies, including chemotherapy and hormone therapy were administered in 90.8% and 70.5% of the patients, respectively, and 67.1% of patients received both treatments. There were 8 of 207 patients (3.8%) who did not receive any adjuvant chemotherapy, endocrine therapy, or irradiation. For Her-2/neu overexpression patients, none of them received adjuvant trastuzumab-based treatment.

### Risk factors for locoregional recurrence, distant recurrence and overall survival

Overall, 16 patients (7.7%) experienced locoregional recurrence: 12 patients recurred on ipsilateral chest wall, two in axillary LN, and the other two patients recurred in supraclavicular LN. The median interval between surgery and locoregional recurrence was 27.9 months (5 to 82 months). Forty of 207 patients (19.3%) developed distant metastasis. Bone, liver and lung metastasis accounted for the most common metastatic sites. Thirty-four patients (16.4%) died during the follow-up.

The relationship between the 5-year LRR, DRR, OS and clinicopathologic characteristics are shown in Table 2. On univariate analysis, young patients (p = 0.003), defined as age less than 40 years, and Her2/neu over-expression (p = 0.016) were significantly related to higher LRR. Presence of LVI was related to higher DRR (p = 0.026). PMRT demonstrated a non-significant, marginal trend of reducing 5-year LRR (from 11.8% to 4.7%, p = 0.1). There were several significant factors correlated with worse 5-year OS, including negativity of ER status (p = 0.033), over-expression of Her-2/neu (p = 0.001) and presence of LVI (p = 0.01). PMRT did not show significance in affecting 5-year OS (p = 0.918).

On multivariate analysis, shown in Table 3, young age patients (HR, 6.53, 95% CI, 1.82–23.38; p = 0.004) and Her-2/neu over-expression (HR, 6.6; 95% CI, 1.79–24.28; p = 0.005) were still associated with higher LRR. Adjuvant chemotherapy, positivity of ER status and LVI showed a non-significant trend of lower incidence of DR. Her-2/neu over-expression (HR, 4.01, 95% CI, 1.64-9.84; p = 0.002) and presence of LVI (HR, 4.99; 95% CI, 1.16–21.55; p = 0.031) were associated with inferior OS significantly.

### Subgroup analysis of LRR and OS in patients treated with or without PMRT

We consequently examined the effect of PMRT on LRR and OS in subgroups. PMRT reduced LR significantly in patients with >25% positive LN (p = 0.033) and in presence of LVI (p = 0.049). Positive LVI was also a predictive marker of better OS if adding PMRT to T1–2 and N1 breast cancer patients (p= 0.047). Although young age and Her2/neu overexpression were independent risk factors of LR, PMRT did not improve LR in such high-risk patients. These results were shown as Table 4.

## Discussion

Curing patients is the paramount goal of treating early breast cancers. Improvement of local-regional control often translates into better survival, not only in eradicating residual local malignant cells but also in reducing distant metastasis.^21^,^22^ The EBCTCG study had analyzed more than 42,000 patients, which showed 19% reduction of 5-year LR risks with PMRT would also reduce 5% risk of 15-year breast cancer mortality.^8^ Although a validated merit of PMRT was confirmed, delayed complications from irradiation including secondary malignancy, cardiac toxicity, lymphedema, skin fibrosis and so on, should be taken into consideration.^41^ Thus, to avoid unnecessary irradiation, it is reasonable to choose patients with high risk of LR to apply PMRT, also to find subgroups of patients who can get benefits from PMRT.

Several predictive markers of LR have been widely discussed. Patients with larger tumor, advanced nodal status, presence of extracapsular extension, positive of LVI, high grade, involvement of the skin, nipple or pectoral fascia, and close or positive resection margins all had been reported to associate with higher risks of recurrence.^14^,^22^–^24^ Therefore, the current consensus by the American Society of Clinical Oncology and other guidelines recommend patients with T3–4 or N2 should receive adjuvant chemotherapy and PMRT definitely if no contraindication.^9^ On the contrast, for patients with T1–2 tumors and 1–3 positive LNs, there are getting more and more debates about whether adjuvant PMRT is needed. The reason of such chaos is because of different intrinsic characteristics of different breast cancers. If we do the Oncotype DX^®^ or MammaPrint^®^ test, we also can see not every patient with positive LN needs adjuvant chemotherapy. But for patients with T1–2 and N1 breast cancer who received total mastectomy, there are still no definite predictive markers for PMRT.

With a retrospective analysis of 8,106 patients enrolled in 13 randomized trials, the 10-year cumulative incidence > 15% for chest wall recurrence in patients with 1–3 positive nodes were age < 40, peritumoral vessel invasion or 0–7 uninvolved nodes. In this study, all patients were PMRT naïve. One of the conclusions proved in patients with 1–3 positive nodes, chest wall PMRT should be considered in patients aged < 40 years, with 0–7 uninvolved nodes or with vascular invasion.^25^ Hunt *et al.* also reported that young age was a risk factor of local recurrence in T1–2 and N0 patients.^26^ In our analysis, the LRR of T1–2 and N1 breast cancer was only 7.7%. Age less than 40 was one of the risk factors of LR, which was compatible with previous reports. Although PMRT did not improve LRR in young age group in our study, small case number might be the major reason of statistic insignificance.

Her-2/neu overexpression, a well-known predictive marker in distant metastasis, is seldom allocated to risk factors of local recurrence from literature review. Currently almost all patients in this group will receive trastuzumab-based adjuvant chemotherapy, which can decrease the chance of distant recurrence but not clearly beneficial on local recurrence. Albert *et al.* retrospectively reviewed 911 T1a-bN0 breast cancer patients who had received definite treatment including surgery and adjuvant chemotherapy. The 8-year LRR were greater in the patients with Her-2/neu-positive (17.5% *vs*. 3.9%, p = 0.009) tumors.^27^ In our study, there were 43 Her-2/neu overexpression patients, and none of them received adjuvant trastuzumab therapy. The reason for lack of adjuvant trastuzumab is that during the period of study enrollment (January 2000 to December 2006), the concept of adjuvant trastuzumab had not been built up. Our study corroborated Her-2/neu-positive tumor was associated with higher LR, however, PMRT failed to add benefits in locoregional control. The reason of radioresistance was supported by preclinical studies; in addition, adding anti-Her-2/neu monoclonal antibody can reverses resistance to irradiation.^28^,^29^

Another risk factor of LR in Karlsson’s study was 0–7 uninvolved LN. Fewer uninvolved LN might be associated with inadequate surgical sampling or pathological examination. Similar result was reported by Duraker *et al.*, who reported fewer removed LN was associated with worse survival.^30^ An indirect method to evaluate the adequacy of removed LN is the ratio of positive LN of all removed LN.^30^–^33^ In our analysis, we also found that PMRT can significantly reduce LR in T1–2, N1 breast cancers with ratio > 0.25 of positive LN. We believe ratio 0.25 can be used as an indicator for PMRT, but it is only suitable for patients who received axillary LN dissection. Besides, there is accumulating data to suggest PMRT, with the coverage of level I–II lymph node areas, can lower the rate of axillary recurrences in patients with positive sentinel LN without LN dissection. This makes PMRT even more important and deservedly.^34^

Lymphovascular invasion has been confirmed as an independent poor prognostic factor in patients with invasive breast cancer.^35^,^36^ The prognostic role of LVI was reported independent of menopausal and LN status, tumor size, tumor grade, or adjuvant treatments. Breast cancers with LVI are candidates for more intensive adjuvant therapies.^20^ Trovo *et al.* analyzed 150 stages I–II breast cancer patients treated with radical mastectomy without adjuvant irradiation. They found statistically significant factors associated with increased risk of LR were premenopausal status (p = 0.004), ER negative (p = 0.02), grade 3 (p = 0.02), and LVI (p = 0.001). They assumed PMRT might be beneficial in patients within these subsets.^37^ In our analysis, we found the presence of LVI significantly related to DR (p=0.026), which also translated to worse OS (p=0.01). Although LVI did not directly related to LR in our report, PMRT could reduce LR in the presence of LVI (p=0.049), just as Trovo *et al.* supposed.

The major debate of PMRT has been focused on whether it should be applied to all T1–2 and N1 breast cancer patients, regardless stratification of high risks. In contrast to DBCG and British Columbia trials, a Japanese study found that PMRT did not offer better locoregional control and OS in patients with 1–3 positive LN who received systemic therapy and adequate dissection.^38^ The recent published study by Yang *et al.* who analyzed 544 T1–2 N1 breast cancer patients with or without PMRT has shown significant reduction of LR and improvement of OS in ER negative and LVI positive patients.^39^ Kyndi *et al.* had analyzed 1,000 of the 3,083 patients in the DBCG 82b & c stratified by ER, PgR and Her-2/neu status. In contrast to Yang’s result, PMRT did not have a survival benefit in ER negative cohort.^40^ Our study examined the effects of PMRT on 207 cases of T1–2, N1 breast cancer patients who received total mastectomy. Although PMRT didn’t influence results of LR and OS in general cohort and may not be routinely applied to be a part of adjuvant treatments, in patients with known risks such as >25% positive LN and LVI present, PMRT certainly reduced locoregional recurrence. Moreover, PMRT significantly improved 5-year OS in LVI positive patients. It makes sense to offer PMRT in selected patients.

Our report possesses several limitations. Firstly, a 5-year observation period is not long enough to precisely predict survival outcome. Secondly, the retrospective nature and small sample size of study have limited statistic power. Due to lack of prospective studies up to now, several large phase III randomized trials are ongoing to solve this issue. The MA 25 study is designed to enroll stage II patients with 1–3 positive nodes treated with radiotherapy versus observation only after mastectomy and adjuvant chemotherapy (NCT00005983). The other trial, SUPREMO study, has been activated recently in order to compare overall survival between PMRT and observation in patients with pT1-2N1 or pT2N0 with histological grade III or LVI positive tumors (NCT00966888). We hope the results will end to long-standing debate.

In conclusion, our work confirmed previous studies that risk factors, including negativity of ER, Her2/neu over-expression, young age and presence of LVI correlated with poor survival outcome and higher locoregional recurrence. In patients with T1–2 and N1 breast cancer, although PMRT by itself is of limited value in establishing locoregional control and OS, it should still be considered in high-risk patients such as with lymphovascular invasion, which will bring on better locoregional control and longer survival.

## Figures and Tables

**TABLE 1.** Clinicopathologic characteristics of patient, tumor and treatment

**Characteristics**	**Radiotherapy**	**No Radiotherapy**	**p value**
No. of patients	81	126	
Age			
Median (years)	50.75	50.43	0.83
< 40	9	20	
≥ 40	72	106	
Histology			
Invasive ductal carcinoma	71	112	0.83
Others	10	14	
Tumor size (T)			
T1	26	48	0.46
T2	55	78	
No. of positive lymph nodes			
1	41	56	0.46
2	21	43	
3	19	27	
Percentage of positive lymph nodes			
< 25%	65	112	0.11
≥ 25%	16	14	
Estrogen receptor status			
Positive	55	82	0.65
Negative	25	43	
Unknown	1	1	
Her-2/neu status			
Over-expressed	19	24	0.72
Not over-expressed	56	84	
Unknown	6	18	
Lymphovascular invasion			
Presence	46	59	0.41
Absence	21	36	
Unknown	14	31	
Adjuvant chemotherapy			
Yes	76	112	0.33
No	5	14	
Adjuvant hormone therapy			
Yes	56	90	0.91
No	24	35	
Unknown	1	1	

PMRT = postmastectomy radiotherapy

**TABLE 2.** Five-year Kaplan-Meier analysis of locoregional recurrence rate, distant recurrence rate and overall survival by basic characteristics of patients and tumors

**Characteristic**	**No. of patients**	**LRR**		**DRR**		**OS**	

		**%**	**P**	**%**	**P**	**%**	**P**
Age (y)			0.003^*^		0.18		0.69
< 40	29	22±9.0%		30.3±9.7%		79.6±8.3%	
≥ 40	178	7±2.2%		17.7±3.1%		85.2±2.9%	
Pathology			0.50		0.52		0.78
Invasive ductal carcinoma	183	9.6±2.5%		18.4±3.2%		84.8±2.9%	
Others	24	5.9±5.7%		26.2±9.3%		81.3±8.6%	
T stage			0.64		0.23		0.27
T1	74	7.7±3.3%		14.0±4.3%		92.4±3.3%	
T2	133	10.1±3.1%		23.8±4.2%		80.2±3.8%	
Numbers of positive LN			0.34		0.22		0.87
1	97	4.7±2.3%		14.3±3.9%		84.8±3.9%	
2	64	15.4±5.4%		22.4±5.5%		85.7±4.7%	
3	46	10.3±4.9%		31.4±8.4%		81.5±6.4%	
% of Positive nodes			0.24		0.77		0.63
< 25%	177	7.7±2.2%		21.1±3.4%		83.1±3.1%	
≥ 25%	30	18.5±8.8%		15.3±7.1%		91.7±5.7%	
ER status			0.25		0.08		0.033^*^
Negative	68	11.0±4.3%		18.8±6.4%		76.8±5.5%	
Positive	137	8.2±2.6%		15.2±3.2%		87.9±3.1%	
Unknown	2						
Her-2/neu			0.016^*^		0.59		0.001^*^
Negative	140	6.2±2.3%		18.8±3.6%		89.2±2.9%	
Positive	43	19.4±6.7%		24.7±7.5%		71.9±7.3%	
Unknown	24						
LVI			0.62		0.026^*^		0.01^*^
Negative	57	6.1±3.4%		3.9±2.7%		96.4±2.5%	
Positive	105	9.7±3.3%		21.1±4.4%		82.7±4.0%	
Unknown	45						
Adjuvant Chemotherapy			0.94		0.16		0.29
No	19	6.2±6.1%		38.1±14.1%		68.9±13.1%	
Yes	188	9.2±2.4%		18.7±3.1%		85.8±2.7%	
PMRT			0.11		0.94		0.92
No	126	11.8±3.2%		20.3±3.9%		83.8±3.5%	
Yes	81	4.7±2.7%		22.9±6.3%		85.6±4.4%	

LRR = locoregional recurrence rate; DRR = distant recurrence rate; OS = overall survival; ER = estrogen receptor; LVI = lymphovascular invasion;

* p < 0.05

**TABLE 3.** Multivariate analysis of locoregional recurrence, distant recurrence and overall survival

**Variable**	**LRR**		**DRR**		**OS**	

	**P**	**HR (95% CI)**	**P**	**HR (95% CI)**	**P**	**HR (95% CI)**
Age (≥ 40 vs. < 40)	0.004^*^	0.15 (0.04–0.55)	NS		NS	
% Positive nodes (>25% vs. ≤25%)	NS (0.064)	3.87 (0.92–16.23)	NS		NS	
ER status (Positive vs. negative)	NS		NS (0.061)	0.45 (0.19–1.04)	NS	
Her-2/neu (Positive vs. negative)	0.005^*^	6.6 (1.8–24.28)	NS		0.002^*^	4.01 (1.63–9.84)
LVI (Positive vs. negative)	NS		NS (0.056)	2.92 (0.97–8.76)	0.031^*^	4.99 (1.16–21.55)
Adjuvant chemotherapy (Yes vs. no)	NS		NS (0.067)	0.36 (0.12–1.08)	NS	
PMRT (Yes vs. no)	NS (0.30)		NS (0.92)		NS (0.23)	

LRR = locoregional recurrence rate; DRR = distant recurrence rate; NS = Non-significant; OS = overall survival; ER = estrogen receptor; LVI = lymphovascular invasion; HR = hazard ratio; CI = confidence interval, PMRT=Postmastectomy radiotherapy;

* p < 0.05

**TABLE 4.** Analysis of clinical benefits on local regional recurrence and overall survival from PMRT

**Characteristic (case numbers)**	**PMRT**	**LRR**		**OS**	

		**%**	**p**	**%**	**p**
Age (y)					
< 40 (29)	No	25	0.6	75	0.37
	Yes	11.1		88.9	
≥ 40 (178)	No	7.5	0.16	84	0.64
	Yes	2.8		84.7	
Pathology					
Invasive ductal carcinoma (183)	No	10.7	0.14	82.1	0.83
	Yes	4.2		85.9	
Others (24)	No	7.1	0.46	85.7	0.4
	Yes	0		80	
T stage					
T1 (74)	No	10.4	0.088	85.4	0.28
	Yes	0		92.3	
T2 (133)	No	10.3	0.42	80.8	0.83
	Yes	5.5		81.8	
Numbers of positive LN					
1 (97)	No	7.1	0.42	85.7	0.77
	Yes	2.4		85.4	
2 (63)	No	11.6	0.82	81.4	0.8
	Yes	9.5		85.7	
3 (46)	No	14.8	0.085	77.8	0.67
	Yes	0		84.2	
% of positive nodes					
< 25% (177)	No	8	0.46	82.1	0.8
	Yes	4.4		86.2	
≥ 25% (30)	No	28.6	0.033^*^	85.7	0.37
	Yes	0		81.3	
ER status					
Negative (68)	No	14	0.24	74.4	0.97
	Yes	4		76	
Positive (137)	No	8.5	0.27	86.6	0.96
	Yes	3.6		89.1	
Her-2/neu status					
Negative (140)	No	7.1	0.43	89.3	0.48
	Yes	3.6		87.5	
Positive (43)	No	25	0.1	58.3	0.45
	Yes	5.3		78.9	
LVI status					
Negative (57)	No	5.6	0.53	97.2	0.24
	Yes	9.5		90.5	
Positive (105)	No	11.9	0.049^*^	72.9	0.047^*^
	Yes	2.2		89.1	

LRR = locoregional recurrence rate; OS = overall survival; ER = estrogen receptor; LVI = lymphovascular invasion; PMRT=Postmastectomy radiotherapy;

* p < 0.05
